# Diaqua­bis­[2-(5-isopropyl-5-methyl-4-oxo-4,5-dihydro-1*H*-imidazol-2-yl)nicotinato]cobalt(II)

**DOI:** 10.1107/S1600536810044715

**Published:** 2010-11-13

**Authors:** Zhong Zhang, Ji-Zhong Liu, Peng Gao, Zhong-Jing Huang

**Affiliations:** a College of Chemistry and Ecological Engineering, Guangxi University for Nationalities, Nanning 530006, People’s Republic of China

## Abstract

In the title complex, [Co(C_13_H_14_N_3_O_3_)_2_(H_2_O)_2_], the Co^II^ atom has a distorted octa­hedral coordination, formed by four N atoms from two (±)-2-(5-isopropyl-5-methyl-4-oxo-4,5-dihydro-1*H*-imidazol-2-yl)nicotinate ligands and two O atoms from two water mol­ecules. Intra­molecular N—H⋯O and O—H⋯O hydrogen bonds are present. In the crystal, inter­molecular O—H⋯O hydrogen bonds link the complex mol­ecules into a chain along [010].

## Related literature

For the synthesis and structures of the compounds containing imidazolidinone derivatives, see: Bombek *et al.* (2005 ▶); Ellis *et al.* (2000 ▶); Erre *et al.* (1998 ▶); Fu *et al.* (2008 ▶). For coordination compounds with pyridine­carb­oxy­lic acids, see: Chatterjee *et al.* (1998 ▶); Nathan & Mai (2000 ▶); Park *et al.* (2007 ▶); Yang *et al.* (2002 ▶).
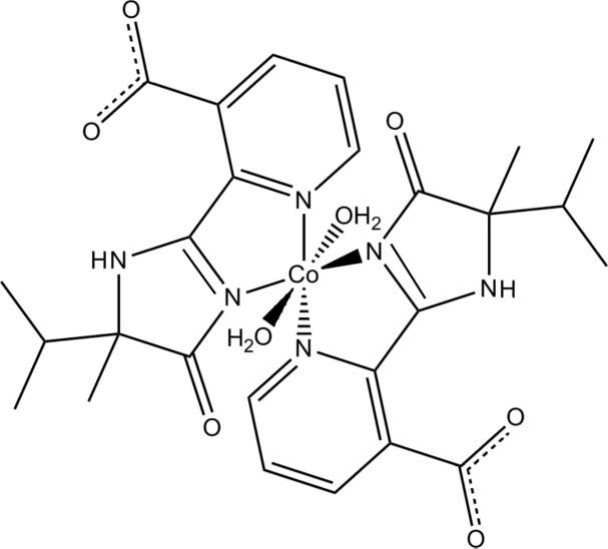


## Experimental

### 

#### Crystal data

[Co(C_13_H_14_N_3_O_3_)_2_(H_2_O)_2_]
*M* *_r_* = 615.51
Orthorhombic, 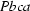

*a* = 12.548 (5) Å
*b* = 19.558 (8) Å
*c* = 23.07 (1) Å
*V* = 5662 (4) Å^3^
*Z* = 8
Mo *K*α radiation
μ = 0.67 mm^−1^
*T* = 298 K
0.43 × 0.42 × 0.40 mm

#### Data collection

Bruker APEX CCD diffractometer
Absorption correction: multi-scan (*SADABS*; Sheldrick, 1996 ▶) *T*_min_ = 0.763, *T*_max_ = 0.777
24468 measured reflections
4992 independent reflections
2623 reflections with *I* > 2σ(*I*)
*R*_int_ = 0.081

#### Refinement

*R*[*F* ^2^ > 2σ(*F* ^2^)] = 0.053
*wR*(*F* ^2^) = 0.142
*S* = 1.03
4992 reflections
376 parameters
2 restraints
H-atom parameters constrained
Δρ_max_ = 0.73 e Å^−3^
Δρ_min_ = −0.42 e Å^−3^

### 

Data collection: *SMART* (Bruker, 2007 ▶); cell refinement: *SAINT* (Bruker, 2007 ▶); data reduction: *SAINT*; program(s) used to solve structure: *SHELXS97* (Sheldrick, 2008 ▶); program(s) used to refine structure: *SHELXL97* (Sheldrick, 2008 ▶); molecular graphics: *DIAMOND* (Brandenburg, 1999 ▶); software used to prepare material for publication: *SHELXTL* (Sheldrick, 2008 ▶).

## Supplementary Material

Crystal structure: contains datablocks I, global. DOI: 10.1107/S1600536810044715/hy2369sup1.cif

Structure factors: contains datablocks I. DOI: 10.1107/S1600536810044715/hy2369Isup2.hkl

Additional supplementary materials:  crystallographic information; 3D view; checkCIF report

## Figures and Tables

**Table 1:** Selected bond lengths (Å)

Co1—N1	2.156 (4)
Co1—N3	2.016 (3)
Co1—N4	2.144 (3)
Co1—N6	2.019 (3)
Co1—O7	2.090 (3)
Co1—O8	2.085 (3)

**Table 2:** Hydrogen-bond geometry (Å, °)

*D*—H⋯*A*	*D*—H	H⋯*A*	*D*⋯*A*	*D*—H⋯*A*
N2—H2⋯O2	0.86	1.75	2.525 (5)	149
N5—H5⋯O5	0.86	1.77	2.537 (5)	148
O7—H7*A*⋯O3	0.85	2.11	2.888 (4)	151
O7—H7*B*⋯O1^i^	0.85	1.81	2.651 (4)	169
O8—H8*A*⋯O6	0.85	2.11	2.857 (4)	147
O8—H8*B*⋯O4^ii^	0.85	1.80	2.645 (4)	170

Symmetry codes: (i) 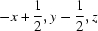; (ii) 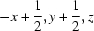.

**Fig. 1.**
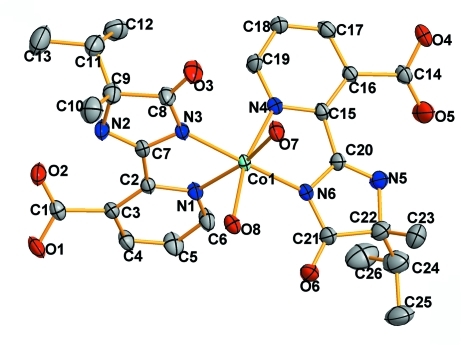
Molecular structure of the title compound. Displacement ellipsoids are drawn at the 50% probability level. H atoms have been omitted for clarity.

**Fig. 2.**
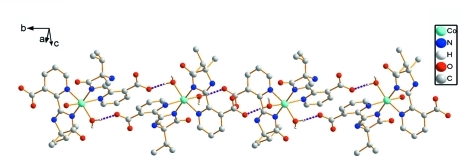
Part of the chain structure in the title compound. Dashed lines indicate hydrongen bonds.

## supplementary crystallographic information

### Comment

Much effort has been devoted to the synthesis (Bombek *et al.*, 2005; Ellis *et al.*, 2000) and crystal structures (Erre *et al.*, 1998; Fu *et al.*, 2008) of the compounds containing imidazolidinone derivatives during the last few years. One of them is (±)-2-(4-isopropyl-4-methyl-5-oxo-4,5-dihydro-1*H*-2-imidazol-2-yl) nicotinic acid (Imazapyr acid), which provides efficient metal-chelating ability. Imazapyr acid containing a pyridinecarboxylic acid and an imidazole ring is well known a versatile ligand. The pyridine carboxylic acid has been extensively used in the design of coordination compounds, due to a variety of bonding modes and ability to form strong hydrogen bonds (Chatterjee *et al.*, 1998; Nathan & Mai, 2000; Park *et al.*, 2007; Yang *et al.*, 2002). Imidazole ring, which is one of the polydentate amine ligands, generally coordinates to metal ions using the N atoms as donors. We report here the structure of the title compound.

The molecular structure of the title complex is shown in Fig. 1. The Co^II^ atom exhibits a distorted octahedral geometry, defined by four N atoms from two imazapyr ligands and two O atoms from water molecules (Table 1). The dihedral angle between the two imazapyr planes in the complex is 64.39 (2)°. Intramolecular O—H···O and N–H···O hydrogen bonds are observed between the coordinated water molecules and imazapyr ligands and between the imidazole and carboxylate groups (Table 2). Intermolecular O—H···O hydrogen bonds link the complex molecules into a chain along [0 1 0] (Fig. 2).

### Experimental

A solution of Imazapyr acid (0.39 g, 1.5 mmol) in 95% ethanol (15 ml) was added dropwise with stirring at room temperature to a solution of Co(CH_3_COO)_2_.4H_2_O (0.13 g, 0.5 mmol) in 20 ml water. The mixture was stirred at room temperature until it was homogeneous and then sealed in a 25 ml Teflon-lined stainless reactor, which was kept under autogenous pressure at 423 K for 5 h and then slowly cooled to room temperature. After 5 days, red block crystals suitable for X-ray diffraction were separated and washed with water. The product is stable in air and insoluble in water and common organic solvents (yield: 76%). Analysis, calculated for C_26_H_32_CoN_6_O_8_: C 50.74, H 5.24, N 13.65%; found: C 50.77, H 5.23, N 13.64%.

### Refinement

H atoms on C and N atoms were positioned geometrically and refined using a riding model, with C—H = 0.93–0.98 Å and N—H = 0.86 Å and with *U*_iso_(H) = 1.2(1.5 for methyl)*U*_eq_(C,*N*). The water H atoms were located in a difference Fourier map and refined as riding atoms, with O—H = 0.85 Å and *U*_iso_(H) = 1.2*U*_eq_(O).

### Crystal data

**Table:**

[Co(C_13_H_14_N_3_O_3_)_2_(H_2_O)_2_]	*F*(000) = 2568
*M**_r_* = 615.51	*D*_x_ = 1.444 Mg m^−^^3^
Orthorhombic, *P**b**c**a*	Mo *K*α radiation, λ = 0.71073 Å
Hall symbol: -P 2ac 2ab	Cell parameters from 1647 reflections
*a* = 12.548 (5) Å	θ = 2.3–19.4°
*b* = 19.558 (8) Å	µ = 0.67 mm^−^^1^
*c* = 23.07 (1) Å	*T* = 298 K
*V* = 5662 (4) Å^3^	Block, red
*Z* = 8	0.43 × 0.42 × 0.40 mm

### Data collection

**Table:**

Bruker APEX CCD diffractometer	4992 independent reflections
Radiation source: fine-focus sealed tube	2623 reflections with *I* > 2σ(*I*)
graphite	*R*_int_ = 0.081
φ and ω scans	θ_max_ = 25.0°, θ_min_ = 1.8°
Absorption correction: multi-scan (*SADABS*; Sheldrick, 1996)	*h* = −14→14
*T*_min_ = 0.763, *T*_max_ = 0.777	*k* = −23→20
24468 measured reflections	*l* = −27→19

### Refinement

**Table:**

Refinement on *F*^2^	Primary atom site location: structure-invariant direct methods
Least-squares matrix: full	Secondary atom site location: difference Fourier map
*R*[*F*^2^ > 2σ(*F*^2^)] = 0.053	Hydrogen site location: inferred from neighbouring sites
*wR*(*F*^2^) = 0.142	H-atom parameters constrained
*S* = 1.03	*w* = 1/[σ^2^(*F*_o_^2^) + (0.0582*P*)^2^] where *P* = (*F*_o_^2^ + 2*F*_c_^2^)/3
4992 reflections	(Δ/σ)_max_ = 0.001
376 parameters	Δρ_max_ = 0.73 e Å^−^^3^
2 restraints	Δρ_min_ = −0.41 e Å^−^^3^

### Fractional atomic coordinates and isotropic or equivalent isotropic displacement parameters (Å^2^)

**Table:**

	*x*	*y*	*z*	*U*_iso_*/*U*_eq_	
Co1	0.27194 (5)	0.10887 (3)	0.37482 (2)	0.03099 (19)	
N1	0.1586 (3)	0.18733 (17)	0.39738 (15)	0.0385 (9)	
N2	0.2182 (3)	0.26551 (19)	0.26096 (15)	0.0527 (11)	
H2	0.1887	0.3050	0.2577	0.063*	
N3	0.2662 (3)	0.16654 (18)	0.30244 (14)	0.0383 (9)	
N4	0.1631 (3)	0.02982 (17)	0.34952 (15)	0.0378 (9)	
N5	0.2214 (4)	−0.0503 (2)	0.48558 (16)	0.0639 (13)	
H5	0.1931	−0.0902	0.4882	0.077*	
N6	0.2623 (3)	0.05068 (18)	0.44694 (15)	0.0382 (9)	
O1	0.0968 (3)	0.42292 (16)	0.37455 (14)	0.0643 (10)	
O2	0.1192 (3)	0.37261 (16)	0.28991 (15)	0.0658 (11)	
O3	0.3576 (3)	0.11596 (18)	0.22656 (14)	0.0684 (11)	
O4	0.1007 (3)	−0.20607 (16)	0.37274 (14)	0.0655 (10)	
O5	0.1140 (4)	−0.15460 (19)	0.45612 (16)	0.0958 (16)	
O6	0.3491 (3)	0.10220 (17)	0.52340 (13)	0.0658 (11)	
O7	0.3937 (2)	0.05098 (15)	0.33722 (13)	0.0502 (9)	
H7A	0.3886	0.0555	0.3007	0.060*	
H7B	0.3991	0.0085	0.3446	0.060*	
O8	0.3882 (2)	0.16846 (14)	0.41510 (12)	0.0480 (9)	
H8A	0.3785	0.1661	0.4515	0.058*	
H8B	0.3928	0.2103	0.4055	0.058*	
C1	0.1092 (4)	0.3720 (2)	0.3438 (2)	0.0420 (12)	
C2	0.1581 (4)	0.2430 (2)	0.36198 (18)	0.0354 (12)	
C3	0.1090 (3)	0.3042 (2)	0.37691 (18)	0.0356 (11)	
C4	0.0523 (4)	0.3037 (2)	0.4295 (2)	0.0482 (13)	
H4	0.0173	0.3431	0.4414	0.058*	
C5	0.0476 (4)	0.2467 (2)	0.4633 (2)	0.0592 (15)	
H5A	0.0074	0.2462	0.4972	0.071*	
C6	0.1035 (4)	0.1901 (2)	0.4461 (2)	0.0534 (14)	
H6	0.1027	0.1517	0.4699	0.064*	
C7	0.2153 (4)	0.2272 (2)	0.30712 (18)	0.0387 (11)	
C8	0.3064 (4)	0.1638 (2)	0.2472 (2)	0.0507 (13)	
C9	0.2802 (5)	0.2306 (3)	0.2150 (2)	0.0572 (13)	
C10	0.3847 (4)	0.2701 (3)	0.2021 (2)	0.0756 (17)	
H10A	0.3681	0.3163	0.1913	0.113*	
H10B	0.4221	0.2481	0.1709	0.113*	
H10C	0.4288	0.2703	0.2361	0.113*	
C11	0.2169 (5)	0.2188 (3)	0.1624 (2)	0.0691 (16)	
H11	0.2597	0.1894	0.1371	0.083*	
C12	0.1139 (4)	0.1788 (3)	0.1769 (2)	0.0757 (18)	
H12A	0.1310	0.1405	0.2012	0.114*	
H12B	0.0817	0.1628	0.1417	0.114*	
H12C	0.0650	0.2084	0.1969	0.114*	
C13	0.1922 (6)	0.2838 (3)	0.1280 (2)	0.113 (3)	
H13A	0.1501	0.2724	0.0947	0.170*	
H13B	0.2577	0.3047	0.1157	0.170*	
H13C	0.1534	0.3152	0.1521	0.170*	
C14	0.1105 (4)	−0.1549 (2)	0.4027 (2)	0.0467 (13)	
C15	0.1611 (3)	−0.0258 (2)	0.38519 (18)	0.0367 (11)	
C16	0.1138 (4)	−0.0873 (2)	0.36856 (18)	0.0369 (11)	
C17	0.0633 (4)	−0.0885 (2)	0.3139 (2)	0.0486 (13)	
H17	0.0308	−0.1284	0.3009	0.058*	
C18	0.0619 (4)	−0.0317 (2)	0.2801 (2)	0.0505 (14)	
H18	0.0264	−0.0320	0.2447	0.061*	
C19	0.1136 (4)	0.0263 (2)	0.2988 (2)	0.0509 (14)	
H19	0.1139	0.0646	0.2749	0.061*	
C20	0.2145 (4)	−0.0108 (2)	0.44084 (18)	0.0418 (12)	
C21	0.3021 (4)	0.0531 (3)	0.5021 (2)	0.0515 (14)	
C22	0.2846 (5)	−0.0172 (3)	0.5315 (2)	0.0660 (15)	
C23	0.3951 (5)	−0.0554 (3)	0.5391 (2)	0.0811 (18)	
H23A	0.3830	−0.1004	0.5543	0.122*	
H23B	0.4394	−0.0301	0.5654	0.122*	
H23C	0.4299	−0.0587	0.5021	0.122*	
C24	0.2291 (6)	−0.0174 (4)	0.5862 (3)	0.094 (2)	
H24	0.2137	−0.0650	0.5966	0.113*	
C25	0.2912 (6)	0.0155 (3)	0.6367 (2)	0.113 (3)	
H25A	0.3569	−0.0087	0.6425	0.169*	
H25B	0.2492	0.0134	0.6714	0.169*	
H25C	0.3063	0.0624	0.6275	0.169*	
C26	0.1250 (6)	0.0190 (4)	0.5779 (3)	0.111 (3)	
H26A	0.1376	0.0673	0.5751	0.167*	
H26B	0.0792	0.0100	0.6104	0.167*	
H26C	0.0916	0.0031	0.5430	0.167*	

### Atomic displacement parameters (Å^2^)

**Table:**

	*U*^11^	*U*^22^	*U*^33^	*U*^12^	*U*^13^	*U*^23^
Co1	0.0399 (3)	0.0183 (3)	0.0348 (3)	−0.0004 (3)	0.0002 (3)	0.0031 (2)
N1	0.044 (2)	0.030 (2)	0.042 (2)	0.0002 (18)	0.0040 (18)	0.0034 (17)
N2	0.079 (3)	0.036 (2)	0.043 (3)	0.020 (2)	0.005 (2)	0.008 (2)
N3	0.051 (2)	0.025 (2)	0.039 (2)	0.0067 (19)	0.0055 (18)	0.0026 (16)
N4	0.045 (3)	0.030 (2)	0.039 (2)	−0.0040 (18)	−0.0031 (19)	0.0024 (17)
N5	0.100 (4)	0.048 (3)	0.044 (3)	−0.034 (3)	−0.010 (2)	0.012 (2)
N6	0.053 (3)	0.025 (2)	0.037 (2)	−0.0035 (19)	−0.0028 (18)	0.0012 (16)
O1	0.100 (3)	0.0241 (19)	0.069 (2)	−0.0034 (19)	0.015 (2)	−0.0037 (17)
O2	0.102 (3)	0.044 (2)	0.052 (2)	0.024 (2)	0.001 (2)	0.0121 (17)
O3	0.098 (3)	0.053 (2)	0.054 (2)	0.026 (2)	0.022 (2)	0.0023 (18)
O4	0.095 (3)	0.027 (2)	0.074 (3)	−0.0002 (19)	−0.009 (2)	0.0005 (18)
O5	0.173 (5)	0.059 (3)	0.055 (3)	−0.057 (3)	−0.006 (3)	0.016 (2)
O6	0.101 (3)	0.046 (2)	0.050 (2)	−0.026 (2)	−0.022 (2)	0.0049 (17)
O7	0.063 (2)	0.0315 (18)	0.056 (2)	0.0090 (16)	0.0096 (17)	0.0037 (15)
O8	0.058 (2)	0.0317 (18)	0.054 (2)	−0.0078 (16)	−0.0026 (16)	0.0035 (15)
C1	0.040 (3)	0.031 (3)	0.055 (3)	0.004 (2)	0.001 (3)	0.002 (2)
C2	0.036 (3)	0.027 (3)	0.043 (3)	−0.001 (2)	−0.004 (2)	0.003 (2)
C3	0.043 (3)	0.026 (2)	0.038 (3)	0.000 (2)	−0.004 (2)	−0.001 (2)
C4	0.058 (3)	0.029 (3)	0.057 (3)	0.005 (2)	0.011 (3)	−0.003 (2)
C5	0.067 (4)	0.050 (4)	0.060 (4)	0.011 (3)	0.026 (3)	0.002 (3)
C6	0.072 (4)	0.035 (3)	0.054 (3)	0.005 (3)	0.020 (3)	0.011 (2)
C7	0.051 (3)	0.027 (3)	0.038 (3)	−0.003 (2)	−0.003 (2)	0.003 (2)
C8	0.070 (4)	0.039 (3)	0.043 (3)	0.014 (3)	0.007 (3)	0.001 (2)
C9	0.072 (4)	0.048 (3)	0.051 (3)	0.008 (3)	0.010 (3)	0.008 (3)
C10	0.077 (4)	0.077 (4)	0.073 (4)	−0.009 (3)	0.013 (3)	0.014 (3)
C11	0.086 (4)	0.066 (4)	0.056 (4)	0.019 (4)	−0.008 (3)	0.002 (3)
C12	0.073 (4)	0.085 (5)	0.069 (4)	−0.009 (4)	−0.018 (3)	−0.004 (3)
C13	0.144 (7)	0.109 (6)	0.088 (5)	0.025 (5)	−0.019 (5)	0.038 (4)
C14	0.053 (3)	0.032 (3)	0.056 (3)	−0.008 (2)	−0.004 (3)	0.004 (3)
C15	0.041 (3)	0.029 (3)	0.041 (3)	−0.005 (2)	0.001 (2)	0.002 (2)
C16	0.044 (3)	0.027 (3)	0.040 (3)	−0.004 (2)	0.002 (2)	−0.001 (2)
C17	0.056 (3)	0.035 (3)	0.056 (3)	−0.008 (2)	−0.001 (3)	−0.006 (2)
C18	0.063 (4)	0.040 (3)	0.048 (3)	−0.006 (3)	−0.018 (3)	0.003 (2)
C19	0.066 (4)	0.040 (3)	0.047 (3)	−0.014 (3)	−0.014 (3)	0.013 (2)
C20	0.056 (3)	0.034 (3)	0.036 (3)	−0.010 (3)	0.000 (2)	0.005 (2)
C21	0.073 (4)	0.040 (3)	0.041 (3)	−0.012 (3)	−0.006 (3)	0.003 (2)
C22	0.094 (4)	0.061 (4)	0.043 (3)	−0.014 (3)	−0.007 (3)	0.004 (3)
C23	0.107 (5)	0.069 (4)	0.067 (4)	0.003 (3)	−0.018 (4)	0.017 (3)
C24	0.120 (6)	0.096 (5)	0.067 (5)	−0.032 (5)	−0.001 (4)	0.002 (4)
C25	0.175 (8)	0.113 (6)	0.051 (4)	−0.036 (5)	−0.016 (4)	−0.008 (4)
C26	0.092 (6)	0.155 (8)	0.086 (5)	0.017 (5)	0.008 (4)	−0.011 (5)

### Geometric parameters (Å, °)

**Table:**

Co1—N1	2.156 (4)	C8—C9	1.539 (6)
Co1—N3	2.016 (3)	C9—C11	1.468 (7)
Co1—N4	2.144 (3)	C9—C10	1.551 (7)
Co1—N6	2.019 (3)	C10—H10A	0.9600
Co1—O7	2.090 (3)	C10—H10B	0.9600
Co1—O8	2.085 (3)	C10—H10C	0.9600
N1—C6	1.321 (5)	C11—C13	1.531 (7)
N1—C2	1.361 (5)	C11—C12	1.548 (7)
N2—C7	1.303 (5)	C11—H11	0.9800
N2—C9	1.482 (6)	C12—H12A	0.9600
N2—H2	0.8600	C12—H12B	0.9600
N3—C7	1.351 (5)	C12—H12C	0.9600
N3—C8	1.371 (5)	C13—H13A	0.9600
N4—C19	1.325 (5)	C13—H13B	0.9600
N4—C15	1.364 (5)	C13—H13C	0.9600
N5—C20	1.292 (5)	C14—C16	1.539 (6)
N5—C22	1.474 (6)	C15—C16	1.396 (5)
N5—H5	0.8600	C15—C20	1.478 (6)
N6—C20	1.351 (5)	C16—C17	1.412 (6)
N6—C21	1.367 (5)	C17—C18	1.357 (6)
O1—C1	1.233 (5)	C17—H17	0.9300
O2—C1	1.249 (5)	C18—C19	1.377 (6)
O3—C8	1.232 (5)	C18—H18	0.9300
O4—C14	1.223 (5)	C19—H19	0.9300
O5—C14	1.233 (5)	C21—C22	1.549 (7)
O6—C21	1.230 (5)	C22—C24	1.442 (7)
O7—H7A	0.8500	C22—C23	1.585 (7)
O7—H7B	0.8500	C23—H23A	0.9600
O8—H8A	0.8500	C23—H23B	0.9600
O8—H8B	0.8500	C23—H23C	0.9600
C1—C3	1.531 (6)	C24—C26	1.500 (8)
C2—C3	1.390 (5)	C24—C25	1.542 (8)
C2—C7	1.487 (6)	C24—H24	0.9800
C3—C4	1.405 (6)	C25—H25A	0.9600
C4—C5	1.363 (6)	C25—H25B	0.9600
C4—H4	0.9300	C25—H25C	0.9600
C5—C6	1.369 (6)	C26—H26A	0.9600
C5—H5A	0.9300	C26—H26B	0.9600
C6—H6	0.9300	C26—H26C	0.9600
			
N3—Co1—N6	174.47 (15)	H10B—C10—H10C	109.5
N3—Co1—O8	94.68 (13)	C9—C11—C13	114.0 (5)
N6—Co1—O8	89.42 (13)	C9—C11—C12	110.6 (4)
N3—Co1—O7	89.18 (13)	C13—C11—C12	111.3 (5)
N6—Co1—O7	94.63 (13)	C9—C11—H11	106.8
O8—Co1—O7	88.65 (12)	C13—C11—H11	106.8
N3—Co1—N4	98.91 (14)	C12—C11—H11	106.8
N6—Co1—N4	77.27 (14)	C11—C12—H12A	109.5
O8—Co1—N4	165.91 (12)	C11—C12—H12B	109.5
O7—Co1—N4	87.84 (13)	H12A—C12—H12B	109.5
N3—Co1—N1	77.18 (14)	C11—C12—H12C	109.5
N6—Co1—N1	99.35 (14)	H12A—C12—H12C	109.5
O8—Co1—N1	87.48 (13)	H12B—C12—H12C	109.5
O7—Co1—N1	165.45 (13)	C11—C13—H13A	109.5
N4—Co1—N1	99.12 (14)	C11—C13—H13B	109.5
C6—N1—C2	118.4 (4)	H13A—C13—H13B	109.5
C6—N1—Co1	125.4 (3)	C11—C13—H13C	109.5
C2—N1—Co1	115.3 (3)	H13A—C13—H13C	109.5
C7—N2—C9	109.6 (4)	H13B—C13—H13C	109.5
C7—N2—H2	125.2	O4—C14—O5	124.9 (5)
C9—N2—H2	125.2	O4—C14—C16	114.6 (4)
C7—N3—C8	106.4 (4)	O5—C14—C16	120.4 (4)
C7—N3—Co1	116.2 (3)	N4—C15—C16	122.0 (4)
C8—N3—Co1	137.3 (3)	N4—C15—C20	111.0 (4)
C19—N4—C15	118.8 (4)	C16—C15—C20	127.1 (4)
C19—N4—Co1	125.2 (3)	C15—C16—C17	116.8 (4)
C15—N4—Co1	115.0 (3)	C15—C16—C14	127.7 (4)
C20—N5—C22	110.3 (4)	C17—C16—C14	115.6 (4)
C20—N5—H5	124.8	C18—C17—C16	120.4 (4)
C22—N5—H5	124.8	C18—C17—H17	119.8
C20—N6—C21	106.8 (4)	C16—C17—H17	119.8
C20—N6—Co1	116.3 (3)	C17—C18—C19	119.2 (5)
C21—N6—Co1	136.5 (3)	C17—C18—H18	120.4
Co1—O7—H7A	107.4	C19—C18—H18	120.4
Co1—O7—H7B	120.3	N4—C19—C18	122.7 (4)
H7A—O7—H7B	107.9	N4—C19—H19	118.6
Co1—O8—H8A	108.1	C18—C19—H19	118.6
Co1—O8—H8B	118.0	N5—C20—N6	114.8 (4)
H8A—O8—H8B	108.6	N5—C20—C15	127.3 (4)
O1—C1—O2	125.3 (4)	N6—C20—C15	117.9 (4)
O1—C1—C3	114.4 (4)	O6—C21—N6	125.0 (4)
O2—C1—C3	120.3 (4)	O6—C21—C22	125.8 (4)
N1—C2—C3	122.8 (4)	N6—C21—C22	109.0 (4)
N1—C2—C7	110.0 (4)	C24—C22—N5	111.6 (5)
C3—C2—C7	127.2 (4)	C24—C22—C21	117.1 (5)
C2—C3—C4	115.6 (4)	N5—C22—C21	98.7 (4)
C2—C3—C1	128.4 (4)	C24—C22—C23	109.0 (5)
C4—C3—C1	116.0 (4)	N5—C22—C23	110.1 (5)
C5—C4—C3	121.5 (4)	C21—C22—C23	110.0 (5)
C5—C4—H4	119.2	C22—C23—H23A	109.5
C3—C4—H4	119.2	C22—C23—H23B	109.5
C4—C5—C6	118.2 (5)	H23A—C23—H23B	109.5
C4—C5—H5A	120.9	C22—C23—H23C	109.5
C6—C5—H5A	120.9	H23A—C23—H23C	109.5
N1—C6—C5	123.2 (5)	H23B—C23—H23C	109.5
N1—C6—H6	118.4	C22—C24—C26	107.9 (6)
C5—C6—H6	118.4	C22—C24—C25	114.5 (6)
N2—C7—N3	115.3 (4)	C26—C24—C25	109.7 (6)
N2—C7—C2	126.1 (4)	C22—C24—H24	108.2
N3—C7—C2	118.6 (4)	C26—C24—H24	108.2
O3—C8—N3	125.5 (4)	C25—C24—H24	108.2
O3—C8—C9	124.7 (4)	C24—C25—H25A	109.5
N3—C8—C9	109.7 (4)	C24—C25—H25B	109.5
C11—C9—N2	112.4 (5)	H25A—C25—H25B	109.5
C11—C9—C8	112.4 (5)	C24—C25—H25C	109.5
N2—C9—C8	99.0 (4)	H25A—C25—H25C	109.5
C11—C9—C10	112.2 (4)	H25B—C25—H25C	109.5
N2—C9—C10	110.6 (4)	C24—C26—H26A	109.5
C8—C9—C10	109.5 (4)	C24—C26—H26B	109.5
C9—C10—H10A	109.5	H26A—C26—H26B	109.5
C9—C10—H10B	109.5	C24—C26—H26C	109.5
H10A—C10—H10B	109.5	H26A—C26—H26C	109.5
C9—C10—H10C	109.5	H26B—C26—H26C	109.5
H10A—C10—H10C	109.5		
			
N3—Co1—N1—C6	175.4 (4)	Co1—N3—C8—O3	5.1 (9)
N6—Co1—N1—C6	−0.2 (4)	C7—N3—C8—C9	1.6 (5)
O8—Co1—N1—C6	−89.2 (4)	Co1—N3—C8—C9	−173.7 (4)
O7—Co1—N1—C6	−164.0 (4)	C7—N2—C9—C11	120.5 (5)
N4—Co1—N1—C6	78.3 (4)	C7—N2—C9—C8	1.6 (5)
N3—Co1—N1—C2	−15.5 (3)	C7—N2—C9—C10	−113.3 (5)
N6—Co1—N1—C2	168.9 (3)	O3—C8—C9—C11	60.4 (7)
O8—Co1—N1—C2	79.9 (3)	N3—C8—C9—C11	−120.8 (5)
O7—Co1—N1—C2	5.2 (7)	O3—C8—C9—N2	179.3 (5)
N4—Co1—N1—C2	−112.6 (3)	N3—C8—C9—N2	−2.0 (5)
O8—Co1—N3—C7	−74.9 (3)	O3—C8—C9—C10	−65.0 (7)
O7—Co1—N3—C7	−163.5 (3)	N3—C8—C9—C10	113.8 (5)
N4—Co1—N3—C7	108.8 (3)	N2—C9—C11—C13	71.2 (6)
N1—Co1—N3—C7	11.4 (3)	C8—C9—C11—C13	−178.1 (5)
O8—Co1—N3—C8	100.0 (5)	C10—C9—C11—C13	−54.2 (7)
O7—Co1—N3—C8	11.5 (5)	N2—C9—C11—C12	−55.1 (6)
N4—Co1—N3—C8	−76.2 (5)	C8—C9—C11—C12	55.6 (6)
N1—Co1—N3—C8	−173.6 (5)	C10—C9—C11—C12	179.5 (5)
N3—Co1—N4—C19	1.0 (4)	C19—N4—C15—C16	3.6 (7)
N6—Co1—N4—C19	176.9 (4)	Co1—N4—C15—C16	−165.4 (3)
O8—Co1—N4—C19	−163.5 (4)	C19—N4—C15—C20	−176.6 (4)
O7—Co1—N4—C19	−87.9 (4)	Co1—N4—C15—C20	14.5 (5)
N1—Co1—N4—C19	79.3 (4)	N4—C15—C16—C17	−2.9 (6)
N3—Co1—N4—C15	169.1 (3)	C20—C15—C16—C17	177.3 (4)
N6—Co1—N4—C15	−14.9 (3)	N4—C15—C16—C14	176.7 (4)
O8—Co1—N4—C15	4.6 (7)	C20—C15—C16—C14	−3.1 (8)
O7—Co1—N4—C15	80.3 (3)	O4—C14—C16—C15	−154.5 (5)
N1—Co1—N4—C15	−112.5 (3)	O5—C14—C16—C15	27.8 (8)
O8—Co1—N6—C20	−163.2 (3)	O4—C14—C16—C17	25.1 (6)
O7—Co1—N6—C20	−74.6 (3)	O5—C14—C16—C17	−152.6 (5)
N4—Co1—N6—C20	12.1 (3)	C15—C16—C17—C18	−0.2 (7)
N1—Co1—N6—C20	109.4 (3)	C14—C16—C17—C18	−179.8 (4)
O8—Co1—N6—C21	8.0 (5)	C16—C17—C18—C19	2.4 (8)
O7—Co1—N6—C21	96.6 (5)	C15—N4—C19—C18	−1.2 (7)
N4—Co1—N6—C21	−176.7 (5)	Co1—N4—C19—C18	166.5 (4)
N1—Co1—N6—C21	−79.4 (5)	C17—C18—C19—N4	−1.7 (8)
C6—N1—C2—C3	4.9 (7)	C22—N5—C20—N6	−2.1 (6)
Co1—N1—C2—C3	−165.0 (3)	C22—N5—C20—C15	178.0 (5)
C6—N1—C2—C7	−174.3 (4)	C21—N6—C20—N5	−2.0 (6)
Co1—N1—C2—C7	15.7 (4)	Co1—N6—C20—N5	171.7 (3)
N1—C2—C3—C4	−4.5 (6)	C21—N6—C20—C15	178.0 (4)
C7—C2—C3—C4	174.6 (4)	Co1—N6—C20—C15	−8.4 (5)
N1—C2—C3—C1	175.0 (4)	N4—C15—C20—N5	175.4 (5)
C7—C2—C3—C1	−5.9 (8)	C16—C15—C20—N5	−4.7 (8)
O1—C1—C3—C2	−153.4 (5)	N4—C15—C20—N6	−4.5 (6)
O2—C1—C3—C2	28.0 (7)	C16—C15—C20—N6	175.3 (4)
O1—C1—C3—C4	26.1 (6)	C20—N6—C21—O6	−178.4 (5)
O2—C1—C3—C4	−152.4 (4)	Co1—N6—C21—O6	9.8 (9)
C2—C3—C4—C5	0.6 (7)	C20—N6—C21—C22	5.0 (6)
C1—C3—C4—C5	−179.0 (5)	Co1—N6—C21—C22	−166.8 (4)
C3—C4—C5—C6	2.8 (8)	C20—N5—C22—C24	128.4 (6)
C2—N1—C6—C5	−1.2 (7)	C20—N5—C22—C21	4.6 (6)
Co1—N1—C6—C5	167.6 (4)	C20—N5—C22—C23	−110.5 (5)
C4—C5—C6—N1	−2.5 (8)	O6—C21—C22—C24	58.0 (8)
C9—N2—C7—N3	−0.8 (6)	N6—C21—C22—C24	−125.5 (5)
C9—N2—C7—C2	−177.9 (4)	O6—C21—C22—N5	177.7 (5)
C8—N3—C7—N2	−0.6 (6)	N6—C21—C22—N5	−5.8 (6)
Co1—N3—C7—N2	175.9 (3)	O6—C21—C22—C23	−67.2 (7)
C8—N3—C7—C2	176.8 (4)	N6—C21—C22—C23	109.4 (5)
Co1—N3—C7—C2	−6.7 (5)	N5—C22—C24—C26	−58.3 (7)
N1—C2—C7—N2	170.6 (4)	C21—C22—C24—C26	54.3 (7)
C3—C2—C7—N2	−8.6 (8)	C23—C22—C24—C26	179.9 (5)
N1—C2—C7—N3	−6.4 (6)	N5—C22—C24—C25	179.2 (5)
C3—C2—C7—N3	174.3 (4)	C21—C22—C24—C25	−68.2 (8)
C7—N3—C8—O3	−179.6 (5)	C23—C22—C24—C25	57.4 (7)

### Hydrogen-bond geometry (Å, °)

**Table:**

*D*—H···*A*	*D*—H	H···*A*	*D*···*A*	*D*—H···*A*
N2—H2···O2	0.86	1.75	2.525 (5)	149
N5—H5···O5	0.86	1.77	2.537 (5)	148
O7—H7A···O3	0.85	2.11	2.888 (4)	151
O7—H7B···O1^i^	0.85	1.81	2.651 (4)	169
O8—H8A···O6	0.85	2.11	2.857 (4)	147
O8—H8B···O4^ii^	0.85	1.80	2.645 (4)	170

Symmetry codes: (i) −*x*+1/2, *y*−1/2, *z*; (ii) −*x*+1/2, *y*+1/2, *z*.
